# Deficits and opportunities, pivots and shifts for scaling-up voluntary medical male circumcision in Uganda: a qualitative reflexive thematic analysis study

**DOI:** 10.1186/s12889-024-19796-w

**Published:** 2024-08-16

**Authors:** John Bekiita Byabagambi, Bruce Hollingsworth, Mark Limmer

**Affiliations:** 1 Department of Health Research, Lancaster University, Kampala, Uganda; 2 Department of Health Research, Lancaster University, Lancaster, UK

**Keywords:** Voluntary medical male circumcision, VMMC, HIV, Deficits, Pivots, Shifts, Reflexive thematic analysis

## Abstract

Despite voluntary medical male circumcision (VMMC) being a cost-effective intervention for preventing HIV transmission, its scale-up has faced challenges. Several interventions to address these challenges in priority countries, including Uganda, have not yielded the desired results. This cross-sectional qualitative study aimed to explore the factors that affect the demand for VMMC and identify possible solutions. Semi-structured phone interviews were conducted with 29 males aged at least 18 and not more than 65 drawn randomly from a database representative of the general population maintained by an independent research organisation. Reflexive thematic analysis was conducted, and data analysis was done using NVivo version 12. The results were presented in narrative format with supporting quotes. The study received ethical and regulatory clearance to be conducted in Uganda. The average age of the respondents was 28 years. Almost all respondents had some education, and most lived in rural areas. Two themes were generated, namely, 1) deficits and opportunities for VMMC, which are issues that currently hinder the uptake of VMMC but, if addressed, would lead to better demand, and 2) pivots and shifts for VMMC, which are changes that need to be made to improve the uptake of VMMC. We found that several challenges, including myths, misconceptions, health system gaps, and uncertainties about the postoperative period, hindered the uptake of VMMC. Pivots and shifts for improving the uptake of VMMC include intensifying VMMC campaigns, addressing inequities, and addressing access barriers. We concluded that several challenges, including myths and misconceptions, health system-related gaps, and uncertainties in the post-circumcision period, persist and negatively impact the scale-up of VMMC in Uganda. VMMC beneficiaries have plausible proposals for addressing challenges. The Uganda Ministry of Health should address the myths, misconceptions, health system-related gaps, and uncertainties about the postoperative period and should involve VMMC beneficiaries in reviewing interventions to address gaps.

## Background

Voluntary medical male circumcision (VMMC) is one of the World Health Organisation-approved methods for the prevention of HIV [1]. VMMC is a package of services that includes health education on HIV prevention, HIV risk-reduction counselling and testing, surgical removal of the foreskin, post-operative education, and review 48 h and seven days post-circumcision. Initial evidence indicates that VMMC reduces the chance of HIV transmission among heterosexual men by approximately 60% [2–4]. Population-based studies have since reaffirmed the effectiveness of VMMC and indicated its HIV protective effect to be as high as 70% [5]. Furthermore, there is evidence to show that VMMC also reduces the chance of HIV transmission among men who have sex with men [6, 7]. The WHO recommends the implementation of VMMC in fifteen priority countries that have a generalised prevalence of HIV either nationally or in some subnational geographies and a low prevalence of circumcision [8]. Between 2008 and 2020, approximately 29.5 million adolescent boys and young men had undergone VMMC, averting approximately 615,000 HIV infections [9]. It is estimated that approximately 4.9 million HIV infections will be averted by 2030 through VMMC [9]. Even with the scale-up of antiretroviral therapy, VMMC is still projected to remain cost-effective in the near future [10, 11].

In addition to HIV prevention among males, VMMC is known to have other benefits [11]. VMMC indirectly benefits women by reducing their exposure to the human papillomavirus, which is known to cause cervical cancer [12]. VMMC also reduces the incidence of other sexually transmitted infections, such as herpes simplex virus type 2, syphilis, and other genital ulcers [13], and there is evidence to show that some men seek VMMC for other benefits beyond HIV prevention and that it acts as a platform for seeking other health services [14, 15].

The scale-up of VMMC has remained challenging across most priority countries, including Uganda. Among the VMMC priority countries, only Kenya, Ethiopia, and Tanzania are on course to reach the targeted 80% saturation [16]. Between 2016 and 2020, 18 million out of a target of 25 million circumcisions were conducted, highlighting the challenges faced with the scale-up [8]. Uganda adopted VMMC as part of its combination HIV prevention strategy in 2010, and by the end of 2020, the coverage of male circumcision was just 57.5% among people aged 15–49 [17]. The scale-up of VMMC in Uganda has faced several challenges, including failure to fully integrate it into existing public health facilities [18], failure to involve women as key stakeholders [19], misconceptions about the sexual benefits of male circumcision [20], and inadequate knowledge [21]. It is still unclear why, despite the long period of VMMC implementation in Uganda and all the interventions implemented to improve the VMMC targets, Uganda remains not on course to meet the WHO targets, and thus, further investigation is required. This study aimed to explore the factors that affect the demand for VMMC and identify possible solutions.

## Methods

### Study design, study participants and setting

This cross-sectional qualitative study was conducted between November 2021 and June 2022 and aimed to recruit males not younger than 18 and not older than 65. Owing to the prevailing COVID-19 down-lockdown restrictions, participants were drawn from a database maintained by an independent research organisation, the International Growth Research and Evaluation Centre (IGREC), based in Uganda [22]. IGREC conducts various types of research, including academic, market surveys, and opinion polls, and maintains a regularly updated database with more than 10,000 respondents representing the general population from all the regions of Uganda. The participants in the database are compensated with phone airtime not exceeding UGX5000 (USD1.38) whenever they participate in a study.

### Data collection

Data collection was conducted virtually through phone interviews to minimise the risk of COVID-19 transmission. Participants were randomly drawn from the database, and if the drawn phone number was unavailable or the participant did not answer their phone, another number would be drawn. At the initial contact, participants were given information about this study and were allowed three days to consider joining. After three days, if they were still interested in joining, the participants gave informed consent before the interview. Before the data collection, the consent information sheet was read to the client in the language they understood best. The participants were allowed to seek clarification, and verbal consent was obtained and documented after determining that they fully understood the study. A semi-structured interview guide was used to conduct interviews in the language best understood by the respondent. The interviews were tape-recorded. The recording only started after the participant’s and interviewer's self-introductions to avoid collecting personally identifiable information. The first author conducted the interviews, assisted by IGREC data collectors who were experienced in virtual data collection and in-depth interviewing and were trained in the research tools. Interviews captured in local languages were translated into English, and a sample was back-translated to check for accuracy by professional translators. All recordings were transcribed manually as opposed to electronic transcription. The recording was permanently deleted after transcription to prevent data from getting into the hands of unauthorised people. Data was stored on a password-protected computer with access only limited to the research team. Interviews were conducted until researchers felt no new information was being generated from additional participants.

### Data analysis

Reflexive thematic data analysis, as described by Braun and Clarke [23]. Data analysis was conducted using NVivo® version 12 (released in March 2020) [24]. Data analysis and collection occurred iteratively. The researchers listened to the recordings to identify emerging topics in the interviews. In addition to the reading that was done during transcription, the transcripts were read several times before and after importation into QSR NVivo® curiously and analytically to gain insight into and become intimately familiar with the collected data. The first reading was done with an open mind to get a general feel and appreciate the richness of the data. In the second reading round, segments of data relevant to the study objective were highlighted noting the language used and emotions such as laughter.

Coding was done manually during the familiarisation stage and electronically using QSR NVivo®, resulting in initial codes being generated. During coding, common data patterns that addressed the same issue about the research question were highlighted with different colours. The process involved moving beyond the apparent meaning of the data and identifying the analytical meaning related to the research question. A two-stage process was used to ensure that no codes were missed and that the codes were mutually exclusive to ensure that each data unit was coded under only one category. The initial codes were used to find semantic or latent pieces of the data that seemed interesting and relevant to the research questions. The initial set of codes reflected the most basic data that could be evaluated effectively. Coding continued until all the data were exhausted and the researchers felt that there were no new codes to be assigned.

The next phase involved moving away from the particular codes to narrow the analysis to a higher level of categories and sub-themes by combining related distinct codes into compositions. Memos describing each sub-theme’s meaning and function were used to record the categorisation process. The validity and reliability of the interpretations were assured by including quotations from respondents taken from the transcripts and used under the appropriate subthemes. Iterative sorting and categorising subthemes yielded preliminary descriptive themes while leaving room for generating other codes and themes. The process of thematic revision sometimes results in consolidation, separation, transformation, dropping vague codes, revising those that do not make sense, and omitting some subthemes.

After creating descriptive sub-themes, the next step in the analysis involved defining the meaning and deciding what data component each sub-theme represented to develop analytical themes. During this phase, several sub-themes were combined or split as appropriate to generate the final themes that were analytical, useful and accurate data representations. The analytical themes were given succinct and straightforward names to give the reader an initial understanding of the topic.

The final phase of the analysis involved drafting a report containing the study’s results. The findings are presented in narratives. Direct quotes from the interviews with the respondents are included in the results; for this, there may be grammatical errors in the excerpts, but these were left to show what the respondents said. Where a respondent used an abbreviation, the full version of the word is indicated in square brackets. Additionally, where keywords were represented by articles such as “it”, “this”, and “them”, the actual referenced word was also included in square brackets.

## Results

Twenty-nine (29) interviews were conducted. The respondents were adult males between 18 and 39 years old, with an average age of 28. Nineteen were single, seven were married, and three were separated. Eighteen were either urban or peri-urban dwellers, while eleven lived in rural areas. Two respondents had no formal education; seven had attended primary school, twelve had attended secondary school, and eight had tertiary-level education.

Two themes, namely, 1) deficits and opportunities for VMMC and 2) pivots and shifts for VMMC, were generated. The first theme regards the issues that hinder the uptake of VMMC. The second theme regards the changes that need to be made to improve the uptake of VMMC. Each major theme has subthemes that are presented in the next section.

### Deficits and opportunities in circumcision

The major theme of deficits and opportunities had four subthemes. These included 1) myths and misconceptions about VMMC, 2) fear of VMMC side effects and impacts, 3) cultural obligations, and 4) health system gaps.

#### Myths and misconceptions about VMMC

Respondents mistrust the government's intentions in promoting VMMC. Several concerns, doubts, and myths about the actual purpose of VMMC hinder the demand for VMMC. Among the concerns is the fear that VMMC is a cover-up for population reduction in Africa. According to some respondents, VMMC is a covert contraceptive that aims to lower the number of black people. *“I have heard of a rumour that the government has the intention of cutting our veins through circumcision to decrease population through the reduction of manpower."* (21-year-old single university graduate from a rural area). *“Propaganda… that the government wants to reduce sexual manpower so that they control the population”* (20-year-old single rural male with tertiary education). *“People say that those injections they use affect you in the future…start feeling like you have electricity. you lose your strength as a man, and you can’t be erect anymore”* (separated 22-year-old rural male and S.4 dropout). Beyond the fear of contraception, some respondents believe that VMMC is a ploy to extract unique valuable chemical strength from the foreskin, which black Africans uniquely possess, reported: *“we Africans have some chemicals that those people don't have, and they do this circumcision looking for that chemical”* (a 26-year-old single diploma holder from an urban area). Respondents cite this as the reason why the foreskin is retained after circumcision.

There are mixed attitudes about VMMC as an HIV prevention intervention. Whereas some respondents believe in VMMC as a trusted HIV prevention intervention, others do not support and do not recognise VMMC as an HIV prevention intervention. Furthermore, some do not recognise it as something that prevents other sexually transmitted infections for historical reasons. The presence of people such as grandparents in the communities who have survived for a long time without being circumcised and without contracting HIV or other diseases is reason enough not to consider VMMC, as mentioned by a 25-year-old single and educated peri-urban male: *“Our fathers grew up, and they were not circumcised, and so I am confident that even if I don’t get circumcised, I will not die (laughs) because it is not a disease not to be circumcised.”*. Additionally, the fact that Muslims and other people who are traditionally circumcised at a very early stage in life can still contract HIV enhances the doubtfulness about VMMC as an HIV prevention intervention. Examples of such opinions included *“I have seen many Muslims who were circumcised when they were still young, but they are [HIV] positive”* (26-year-old single diploma holder from an urban area).

Personally, I think that it has less to do with HIV prevention because even if you are circumcised, and you are having intercourse, you can still get HIV if your partner is infected. Therefore, I truly don’t see how circumcision can prevent HIV (27-year-old single urban male).

Contrary to its intended purpose, some respondents view circumcision as enhancing the chances of acquiring HIV. There is a belief that circumcision may aid the transmission of HIV through cultural practices. In some cultures, there is a belief that circumcision comes with a bad omen that is transferred to the first female that one has intercourse with post-circumcision. To avoid transmitting the curse to the spouse, newly circumcised men are forced to find other sexual partners, increasing their chance of acquiring HIV by having multiple sexual partners. Among the voices that supported this were:

There is propaganda that if the man is circumcised and married, after healing, the first person he should have sexual intercourse with should not be his wife because it means a bad omen for her. From then on, she started discouraging me after hearing that myth; she is suspicious of what would happen after my circumcision (37-year-old single certificate holder).

There is a need to address risk compensation after circumcision. The thought that, after circumcision, one is immune to HIV infection is another reason why some people do not recommend VMMC for their loved ones. This was mentioned by a 29-year-old single university graduate and rural male who said that *“some of them are totally misled because they think that once one is circumcised, they don’t get HIV at all, so they engage carelessly with anyone, and this mentality is not good, so to get rid of that, they need to be educated.”*

Similar to its HIV prevention role, the impact of circumcision on sexual performance and pleasure evokes mixed reactions. The uncertainty about the implications of VMMC on sexual performance renders VMMC not an option for some respondents. *“You could be used to a different experience during sex when you are not circumcised, and then when you get circumcised, you get a totally different and bad experience, and it makes you wonder why you got circumcised”* (28-year-old single peri-urban male). Whereas some people experience positive outcomes, testimonies from people who share negative sexual experiences post-circumcision discourage other would-be seekers of VMMC. *“We hear stories from people who have been circumcised that they lost manpower, so others also fear losing their manpower because of circumcision, and they prefer to do without it (laughs)”* (26-year-old single diploma holder and urban dweller).

The conflict between VMMC and religion is another reason respondents are against VMMC. Some respondents associate circumcision with Islam and worry that VMMC is used to initiate and convert them to Islam. Since they are not willing to change their religion, they are not willing to consider VMMC, as stated by a 31-year-old single and educated rural male: *“The way I see it, most people are not educated on the importance of circumcision because the Muslims sometimes come, but people have perspectives and think that they want to make them change their religion to Islam”.*

#### Fear of VMMC side effects and impacts

The poor management of post-circumcision pain and the absence of explicit knowledge and information on post-circumcision care and what to expect are other hindrances to the uptake of VMMC. One respondent who shared information about their post-circumcision period said, *“I got cured, but the pain I went through, I regretted”* (30-year-old married certificate holder from a rural area). Furthermore, there is ignorance and a lack of clarity about how post-circumcision pain is prevented and managed, and this creates room for people to imagine all sorts of things about wound care. One respondent shared their perspective on these thoughts as presented below.

I am told that after circumcision, they stitch you with gauze, so for someone to stitch you with gauze when you have a normal size, what happens when you are erect, yet the gauze was tied when you were down? And yet, we all know that when you are erect, the size expands (laughs). It is equivalent to tying a wire on a tree, and the tree grows, making the wire enter the tree, so when I think about all those things and the idea of the pain I will go through, including waking up while crying, I can’t handle it (a 26-year-old single diploma holder, urban).

#### Cultural obligations

The strong desire to meet subjective cultural norms and expectations supersedes the need to seek safe medical circumcision, thus driving men to shun VMMC and seek non-medical circumcision. Men from cultures that practice traditional circumcision avoid VMMC because of the belief that a man who is not culturally circumcised is considered a coward and may be subjected to cultural circumcision even after undergoing VMMC. Furthermore, the cultural belief that when a portion of one’s body part is buried without performing cultural rituals while the person is still alive, it brings a bad omen is another reason to seek traditional male circumcision (performed outside a medical setting as part of the rite of passage from childhood into adulthood) instead of medical circumcision, as was shared by one of the respondents.

Another issue that is so scary is that in the case of the Bamasaba [tribe in Uganda], the culture that practices circumcision, they have a saying that if a person goes through SMC [safe male circumcision], he has to do it again, that they go and they circumcise him according to their culture because they do not believe in SMC; therefore, someone going through double the pain and payment is my worry (a 31-year-old married male and certificate holder from a rural area).

#### Health system gaps

Low confidence in service providers is another reason that negatively affects the uptake of VMMC. While circumcision within a healthcare setting such as a hospital is considered safe and better than traditional circumcision, several respondents cited the use of junior health workers as a deterrent to undergoing VMMC. Several respondents raised this concern, including the following: *“The doctor who worked on us was in training, and it worried me because I didn't have the confidence that he would do it well, but surprisingly, all went well”* (18-year-old single S.4 student, urban male). Another participant noted, *not getting a good and qualified health worker to work on you, and they do the procedure badly”* (38-year-old single and S.1 dropout urban dweller). Furthermore, there is a belief that mosques have better and more experienced circumcisers than health units, hence the reason to seek religious and traditional circumcisions and not VMMC.

For me, I told myself that I would get a Muslim, and I already saw a number of people at the mosque; he would find a place to circumcise me, then I would go to the pharmacy, and they would prescribe drugs for me that I would buy. However, going to those camps, you find university students who are in internships, and I have a friend whose vein was cut recently. They had to fly him out of the country to treat him, so those are some of the things that discourage me because sometimes they use untrained people (26-year-old single diploma holder, urban dweller).

Low confidence in the health care system and the public health approach used to offer VMMC are other gaps that must be addressed. The provision of VMMC services at camps appears to hinder service uptake. The absence of privacy during surgical camp circumcisions, where young boys are circumcised in the same rooms as adults, discourages older people (*“young boys and old men were circumcised in the same room)”* (32-year-old single and educated urban male) and the doubt about the quality of drugs used in the camps (*“the drugs they give people in those mobilised camps are not the same type as the ones they give in the pharmacies and drug shops”)* (26-year-old single diploma holder, urban male), are other reasons why people shun VMMC.

Furthermore, the fear of being tested for HIV, as part of the VMMC packages, is another hindrance to the uptake of VMMC. People do not want to be tested for HIV since they may not be ready for it, as mentioned by a 21-year-old single university graduate from a rural area: *“People are tested for HIV when they are not ready for it.”*

### Pivots and shifts for VMMC

The second theme created was pivots and shifts for VMMC. This theme included the considerations and adjustments needed to improve the demand for VMMC in Uganda. They included 1) increasing VMMC sensitisation campaigns, 2) highlighting other benefits of circumcision beyond HIV prevention, 3) addressing inequities, and 4) addressing fears related to circumcision.

#### Increase VMMC sensitisation campaigns

To address myths, concerns, and other inaccurate beliefs about VMMC, respondents recommended that more VMMC sensitisation campaigns be conducted to reach all communities: *“Campaigns to reach as many people as possible”* (27-year-old single urban male) and *“sensitisation among the communities”* (21-year-old separated primary school dropout from an urban area). The messaging should target not only prospective clients but also people who positively influence men, such as sexual partners and school head teachers. It is recommended that the messages be delivered by trusted people, such as village health teams. Examples of suggestions shared included *“Campaigns in the villages… and in towns [are required] because people need to be given information before they are circumcised”* (22-year-old separated S.4 dropout and rural male); *“There should be enough sensitisation for the parents and the clients”* (21-year-old single university graduate from a rural area); *“Start with schools because that is where most teenagers are”* (20-year-old single and rural male with tertiary education); *“The VHTs [village health teams] should educate people mostly in rural places at the grassroots to increase awareness”* (21-year-old single university graduate from a rural area); and *“Start at the national level, and the government should prioritise sensitisation. Inform people and put those things on the radio and let them put banners up so that people can get clear information that indicates that you go to this place.”* (32-year-old single and educated urban male).

#### Highlight other benefits of circumcision beyond HIV prevention

There is a need to sensitise people about other benefits of VMMC since HIV prevention is not a compelling reason to convince some people to undergo VMMC. Examples of recommendations on the benefits of circumcision were shared, as follows:

I think it is a good thing ……., there is another training that I also attended, and they said that it helps to reduce the spread of cancer among women. That those men who are not circumcised in the skin around the neck, there are those viruses of cervical cancer, so those people who are not circumcised spread that virus to women because we men are carriers since we don't have a cervix, but we spread to women, so circumcision helps with that, as well. Therefore, people should be informed about this benefit (31-year-old married and educated rural male).

“It works. Any man should be clean, and if you are not circumcised, there is dirt you keep inside. I went for a circumcision last year during COVID because every time I would go to bathe, I would see dirt inside me, and it is a bad thing, but when you get circumcised, all that dirt goes away, so it gave me the courage to go and get circumcised, and since I was circumcised, I feel a lot of peace, and I am fine.” (20-year-old single and educated peri-urban male).

#### Address inequities

Addressing inequities in VMMC service delivery is important. Financial expenses associated with VMMC service delivery impede some people, such as young people, from seeking VMMC. Whereas the actual circumcision service is free, circumcised people incur expenses to access post-circumcision care in the form of medical fees, transportation costs, and missed earnings while away from their duties to seek post-VMMC care. One of the respondents recommended that *“they should encourage circumcision, and it should be free of charge; however, there should also be additional support to help those who undergo VMMC, like financial support.”* (a 30-year-old married certificate holder from a rural area). Another respondent said,

"If we are looking at young people, teenagers, and young adults, especially those who are uneducated, it would be hard for them to pay for these services, so I think it would be a better thing for them to make the service free of charge." (a 32-year-old, single, and educated urban male).

The financial barriers notwithstanding, there is a need to improve geographical access and have flexibility and regularity in service delivery to support the scale-up of VMMC. Respondents recommended that VMMC services be brought closer to where people who need the services are located and that VMMC sites be responsive to the needs of the people; [clinics] *“are very far, and this has hindered most of our teenagers to go and undergo [VMMC]”* (20-year-old single and rural male with tertiary education). Flexibility in service hours, such as opening after hours and over the weekend, to cater to people who may not be able to attend during the typical 8 am to 5 pm period, is also important. Furthermore, respondents recommended regularity in VMMC service delivery to aid people in planning when to seek services. Examples of the voices that supported these recommendations included the following: *“Circumcision should be continuous in our health centre because most of our peers are not willing to move”* (31-year-old married certificate holder from a rural area), and *“they should also increase the number of circumcision sites within the communities because the health facilities may be far and this voluntary circumcision doesn’t happen every day”* (22-year-old separated and S.4 dropout rural male).

#### Address fears related to circumcision

Addressing all the misconceptions about VMMC, including the concern that circumcision is conducted without anaesthesia and that men are required to wear skirts during healing after circumcision. Respondents recommended informing people that *“there is sterilising [anaesthesia] because most of them fear going through the pain”* and informing people that *“someone can still put on his pants and go to work even after circumcision because that is their greatest fear”* (31-year-old married certificate holder from a rural area). Another respondent recommended that people be informed that *“one does not need a skirt after circumcision”* (20-year-old single, less educated, peri-urban youth).

## Discussion

Despite VMMC being a cost-effective intervention, countries like Uganda have failed to achieve the UNAIDS fast-track targets to reach 80% VMMC saturation among males aged between 15 and 49 [25]. The current study explored the factors that affect the demand for VMMC and identified possible solutions. It generated two major themes. First, deficits and opportunities for VMMC are issues that currently hinder the uptake of VMMC, but if addressed, they would lead to better demand for VMMC. The deficits and opportunities for the VMMC theme had four sub-themes: 1) myths and misconceptions about VMMC, 2) fear of VMMC side effects and impacts, 3) cultural obligations, and 4) health system gaps. The second major theme generated was about pivots and shifts for VMMC, which are changes that should be made to improve the uptake of VMMC. This major theme had the following subthemes. 1) increasing sensitisation campaigns, 2) highlighting the benefits of circumcision other than HIV prevention, 3) addressing inequities, and 4) addressing fears related to circumcision. The pivots and shifts are not necessarily direct solutions for the deficits and opportunities.

The current study's findings align with those identified in a systematic review of barriers and facilitators of VMMC that assessed studies published before 2016 [26]. Astonishingly, some of these challenges have persisted for several years since this evidence was available, which may partially explain the failure to meet the VMMC targets.

The challenges that pertain to the myths and misconceptions about VMMC identified in this study include VMMC being considered a disguised form of contraception, the fear that VMMC negatively affects sexual performance and that VMMC is aimed at converting Christians into Muslims. Nanteza et al. [21] found that misconceptions about the negative impact of VMMC on sexual performance were associated with a lower prevalence of VMMC in northern Uganda. In a study conducted among commercial motorcycle riders in western Uganda, Tusabe et al. [20] found that the fear of being converted to Islam was one of the factors hindering the uptake of VMMC. Myths and concerns about VMMC are not unique to Uganda. Moyo et al. [27] identified similar myths in Zimbabwe. Despite several interventions to address the misconceptions about VMMC in Uganda that have been implemented [28], the misconceptions persist and continue to impede the scale-up of the service. In the current study, respondents believe that the information about VMMC is still inadequate, and thus, there is a need to reach communities with appropriate messaging. Sgaier et al. [28] recommend using a multifaceted approach that involves tailored messages and reaching out to traditional leaders to act as advocates for the program, while Byaruhanga et al. [19] recommend the involvement of women as a strategy to address misconceptions about VMMC. The existing approaches for addressing VMMC misconceptions must be reviewed to understand what, where, who, and how they should be implemented.

Participants in the current study recommend the addition of other benefits of circumcision beyond HIV prevention to VMMC community-based messaging as a way of attracting people to seek VMMC. Whereas this might improve uptake, it may not address the primary purpose of HIV prevention if circumcised individuals do not abandon risky HIV behaviours since the reason for seeking circumcision is not HIV prevention. VMMC programs need to cautiously provide messaging on other benefits of VMMC beyond HIV prevention. Whereas messaging on additional benefits will help improve the uptake of medical circumcision, it should not be done in isolation but should be combined with HIV risk reduction messaging. The messaging should preferably be done using approaches that provide real-time feedback that allows for seeking clarifications [29]. Zulu and Mwamba [30] recommend leveraging community structures and key stakeholders, who are often locally trusted sources of information, to convey messages about VMMC.

Health system gaps are another hindrance for VMMC. This study finds that men do not like the current approach of using surgical camps that offer VMMC services. There is mistrust in the drugs used in surgical camps, as they are considered substandard, and there is also a lack of confidence in the service providers in surgical camps. To scale up VMMC service delivery, the Uganda Ministry of Health offers VMMC services through surgical camps, typically involving intensified mobilisation of people for circumcision followed by high-volume circumcisions. This is part of the “Models for Optimising Volume and Efficiencies” (MOVE) guidelines [31].

Similar to what Nxumalo and Mchunu [32] found in Kwa Zulu Natal, one of the concerns identified in this study was a lack of confidence in service providers. Whereas in the current study, we could not tell if the queried service providers were qualified health workers, Matumaini et al. [33] found no difference in the rate of adverse events regarding circumcisions conducted by medical doctors and those undertaken by non-doctor health workers. One respondent reported preferring to be circumcised at a mosque to a hospital, further underscoring the preference for hands-on experience for safety. Task shifting the role of performing medical circumcision from medical doctors to other health professionals, such as nurses and clinic officers or associates, is a recommended strategy for scaling up VMMC, given the shortage of medical doctors in several VMMC priority countries [34, 35].

A particular concern was raised about the lack of privacy in circumcision camps. Assuring privacy needs to be urgently addressed if the program is to reach the desired pivot among 15–29-year-olds and other older males. Before opting for the camp model of providing VMMC, all mixed opportunities for integrating VMMC into outpatient services at health facilities should be exhaustively explored and utilised [36].

The current study identifies the other category of deficits and opportunities as fears and concerns post-circumcision. Among these are the perceived fear of pain, adverse events, and associated costs for managing the adverse events. Whereas the fear of pain and actual pain during circumcision is a well-documented concern [26, 37], it unfortunately remains unaddressed. The fear of pain remains one of the biggest concerns among people seeking male circumcision [38]. Respondents in the current study recommended further sensitization and letting prospective clients know that the procedure is done under anaesthesia and thus painless. This recommendation speaks to the importance of addressing the why, how, and where of VMMC during community mobilisations.

Contrary to its intended benefit, it is surprising that some respondents think VMMC might aid HIV transmission. Respondents cited the cultural requirement of having sexual intercourse with another woman to avoid transferring bad omens to the spouse as a way in which VMMC may aid HIV transmission. Kibira et al. [39] have previously documented the concern about the need to find another sexual partner. It is encouraging to know that people still think about HIV transmission even after circumcision because this implies that they understand the limits of VMMC and HIV prevention interventions. Although HIV risk compensation has not been identified among circumcised clients [40–42], it needs to be continuously monitored so that it can be addressed should it emerge.

This study has limitations because it only explored the demand side of VMMC and did not explore the challenges and possible solutions from VMMC service providers. As noted by Kiyai et al. [18]. There are several missed opportunities from the supply side for scaling up VMMC, which could be the key to meeting the VMMC targets. Further, the study participants were not directly drawn from the general population but from a compiled database. They may not fully represent the general population.

## Conclusions

Despite VMMC being a cost-effective HIV prevention intervention, its scale-up remains challenging. Perceived barriers such as myths and misconceptions, fear of what happens post-circumcision, and health system-related challenges such as distant VMMC clinics continue to hinder its scale-up. Adult males have proposals for addressing the challenges that should be further explored by the Uganda Ministry of Health and other stakeholders supporting VMMC. In addressing these challenges, considerations about who, where, when, and how the possible solutions are implemented are crucial in remediating the challenges.

## Data Availability

The datasets used and/or analysed during the current study are available from the corresponding author on reasonable request.

## Abbreviations

COVID-19: Coronavirus Disease of 2019
HIV: Human immunodeficiency virus
MOVE: Models for Optimising Volume and Efficiencies
VHT: Village Health Team
VMMC: Voluntary medical male circumcision
