# Correction: Deficits and opportunities, pivots and shifts for scaling-up voluntary medical male circumcision in Uganda: a qualitative reflexive thematic analysis study

**DOI:** 10.1186/s12889-024-19845-4

**Published:** 2024-08-26

**Authors:** John Bekiita Byabagambi, Bruce Hollingsworth, Mark Limmer

**Affiliations:** 1 Department of Health Research, Lancaster University, Kampala, Uganda; 2 Department of Health Research, Lancaster University, Lancaster, UK

**Correction: BMC Public Health (2024) 24:2232**

10.1186/s12889-024-19796-w

The original publication of this article contained an incorrect order of author names. The incorrect and correct information is listed in this correction article, the original article has been updated.

Incorrect

Byabagambi Bekiita John, Hollingsworth Bruce, and Limmer Mark

Correct

John Bekiita Byabagambi, Bruce Hollingsworth and Mark Limmer

